# Identification and Genetic Analysis of Downy Mildew Resistance in Intraspecific Hybrids of *Vitis vinifera* L.

**DOI:** 10.3390/plants14152415

**Published:** 2025-08-04

**Authors:** Xing Han, Yihan Li, Zhilei Wang, Zebin Li, Nanyang Li, Hua Li, Xinyao Duan

**Affiliations:** 1 Shandong Academy of Grape, Shandong Academy of Agricultural Sciences, Jinan 250100, China; hanxing@nwafu.edu.cn; 2 College of Enology, Northwest A&F University, Yangling, Xianyang 712100, China; liyihan@nwafu.edu.cn; 3 College of Enology and Horticulture, Ningxia University, Yinchuan 750021, China; wangzl1994@nxu.edu.cn; 4 School of Landscape and Ecological Engineering, Hebei University of Engineering, Handan 056038, China; lizebin198@163.com

**Keywords:** *Vitis vinifera* L., intraspecific hybridization, downy mildew, inheritance of disease resistance

## Abstract

Downy mildew caused by *Plasmopara viticola* is an important disease in grape production, particularly in the highly susceptible, widely cultivated *Vitis vinifera* L. Breeding for disease resistance is an effective solution, and *V. vinifera* intraspecific crosses can yield progeny with both disease resistance and high quality. To assess the potential of intraspecific recurrent selection in *V. vinifera* (IRSV) in improving grapevine resistance to downy mildew and to analyze the pattern of disease resistance inheritance, the disease-resistant variety Ecolly was selected as one of the parents and crossed with Cabernet Sauvignon, Marselan, and Dunkelfelder, respectively, creating three reciprocal combinations, resulting in 1657 hybrid F1 progenies. The primary results are as follows: (1) significant differences in disease resistance among grape varieties and, significant differences in disease resistance between different vintages of the same variety were found; (2) the leaf downy mildew resistance levels of F1 progeny of different hybrid combinations conformed to a skewed normal distribution and showed some maternal dominance; (3) the degree of leaf bulbous elevation was negatively correlated with the level of leaf downy mildew resistance, and the correlation coefficient with the level of field resistance was higher; (4) five progenies with higher levels of both field and in vitro disease resistance were obtained. Intraspecific hybridization can improve the disease resistance of offspring through super-parent genetic effects, and Ecolly can be used as breeding material for recurrent hybridization to obtain highly resistant varieties.

## 1. Introduction

Grapes represent a significant fruit crop worldwide, particularly for wine-producing countries [1]. The International Organization of Vine (OIV) reported that China hah a viticulture area reaching 755,000 hectares in 2023, ranking it third globally. Despite the notable achievements of its grape and wine industry, the excellent viticulture areas of China are mainly distributed in the arid and semi-arid regions of northern China. Owing to the distinct continental monsoon climate, these regions have hot and rainy summers; hence, the severe fungal diseases during grape growth, especially the occurrence of downy mildew, affect the photosynthesis of leaves, reduce fruit quality, and lead to a decrease in yield [2]. In response, traditional chemical control methods are employed. However, this approach not only increases production costs and causes environmental pollution and fruit residue but also accelerates the mutation of pathogens, resulting in the emergence of new types of disease resistance [3]. These problems can be addressed, and sustainable development of viticulture can be realized by breeding new grape varieties or strains with high quality and disease resistance, thus promoting the development of the grape and wine industry in China.

In the main grape-producing areas, breeders worldwide have worked to select new varieties with high disease resistance to adapt to the local ecological environment [4,5,6,7]. However, the development of the grape industry remains largely limited by the lack of quality varieties [8]. The selection and breeding of new varieties can be most widely and effectively implemented by cross-breeding as it can quickly integrate the desired genes of many varieties to obtain new varieties with enhanced characteristics [9]. Interspecific hybridization has led to significant advances in grape disease resistance breeding [10,11,12]. Using this method, breeders have selected the variety ‘A. Bouquet’, which is resistant to downy mildew and powdery mildew [13], and the variety ‘Itasca’, which is resistant to downy mildew, powdery mildew, and pests [14]. These interspecific hybrids typically exhibit high disease resistance but tend to have low yields and produce wines with undesirable organoleptic characteristics, including high acidity, low astringency, overly herbaceous flavours, or undesirable aromas [11,15,16]. Intraspecific hybridization has markedly advanced efforts to improve grape fruit quality. The red variety Cabernet Sauvignon [17] and the white variety Chardonnay [18], widely cultivated globally, were selected through intraspecific hybridization. These crosses possess flavour profiles necessary for making quality wines but are susceptible to pests, diseases, and extreme temperatures [11,19,20,21].

*V. vinifera* varieties are typically of high grape quality and sensitive to fungal disease, but there are still differences in their resistance among different varieties, indicating that the *V. vinifera* varieties themselves possess micro-effect polygenes for disease resistance. In intraspecific recurrent selection in *V. vinifera* (IRSV), high-quality *V. vinifera* varieties were used as parents, and the micro-effect disease resistance genes from *V. vinifera* accumulated within the same genotype via gene substitution between genes through intraspecific recurrent selection, significantly improving the disease resistance of new materials [22]. Thus, IRSV can be an effective approach to breeding grapes with high quality and disease resistance, and the Ecolly and Meili varieties have been successfully bred using this method [23,24]. However, the unclear pattern of disease resistance inheritance in *V. vinifera* intraspecific hybrid progeny has significantly hindered parental selection and early screening.

In this study, the high-quality, disease-resistant variety Ecolly was selected as the parent and subsequently crossed with three high-quality varieties—Cabernet Sauvignon, Dunkelfelder, and Marselan—to obtain F1 intraspecific hybrid progeny. The degree of leaf bulbous elevation, field disease index (DI), and in vitro DI for each F1 progeny from six cross combinations were determined over two consecutive years. The disease resistance of each F1 progeny was graded to assess the potential of the hybrid strains derived from Ecolly in enhancing the resistance of grape to downy mildew. This study initially revealed the genetic characteristics and distributions of disease resistance within *V. vinifera* intraspecific hybrid progeny. This research provides a basis for parental selection and progeny identification in IRSV.

## 2. Results

### 2.1. Identification of Disease Resistance of Hybrid Parents

**Figure 1:**
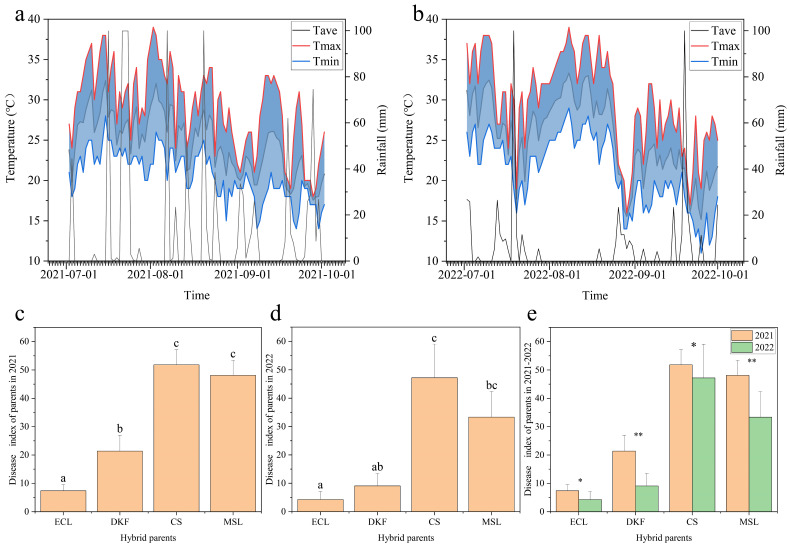
Daily temperature, rainfall, and field downy mildew disease index (DI) for the four parents during the growing season. Note: (**a**,**b**): Daily average temperature, daily maximum temperature, daily minimum temperature, and daily rainfall for July, August, and September during the 2021 and 2022 growing seasons; (**c**,**d**): Field downy mildew DI of four parents at peak disease period in 2021 and 2022. Different letters indicate a significant difference between varieties as determined by Duncan’s multiple range test (*p* ≤ 0.05); (**e**): Field epidemic index of four parents at peak disease incidence in different years. “*” denote a significant difference between varieties as determined by Duncan’s multiple range test (*p* ≤ 0.05), “**” denote a extremely significant difference (*p* ≤ 0.01).

As shown in Figure 1, a significant difference in rainfall in Yangling during July, August, and September was observed between 2021 and 2022. The most considerable difference was observed in August, with 2022 receiving substantially less rainfall than the previous year. No significant difference in high and low temperatures was observed for July and August between the two years. The average temperature predominantly fell between 20 °C and 30 °C. Significant differences in the field downy mildew DI of the same parents at peak disease period were evident between the two years. Specifically, the DI of the four parents in 2022 was generally lower than in 2021, consistent with the rainfall trend. In addition, significant differences in the field downy mildew DI were observed among the four parents at the peak disease period within the same year. The DI ranking was consistent across years, as follows: Cabernet Sauvignon > Marselan > Dunkelfelder > Ecolly.

### 2.2. Identification and Genetic Analysis of Disease Resistance in V. vinifera Hybrids

Bias statistics and genetic analyses were conducted over two years on the field downy mildew DI in offspring populations of six hybrid combinations, across two batches (Table 1). Significant differences in the bias of positive and negative crossbred progeny groups were observed for each hybrid combination across different years. The DI of progeny from combinations with Ecolly as the maternal parent was significantly higher than that of progeny from combination with Ecolly as the paternal parent. Analysis of the field downy mildew DI indicated significant differences in the coefficient of variation (CV) among different hybrid combinations. Specifically, 2022B exhibited the highest CV, from 61.90% to 84.26%. The generalized heritability (H^2^) for the DI in different hybrid combinations was relatively stable, with 2022B exhibiting the highest H^2^. In both 2021 and 2022, the C1 combinations exhibited the highest ultra-high affinity ratio (HH). These results demonstrate strong maternal effects and quantitative inheritance, with Ecolly contributing significantly to resistance in most hybrid combinations.

**Figure 2:**
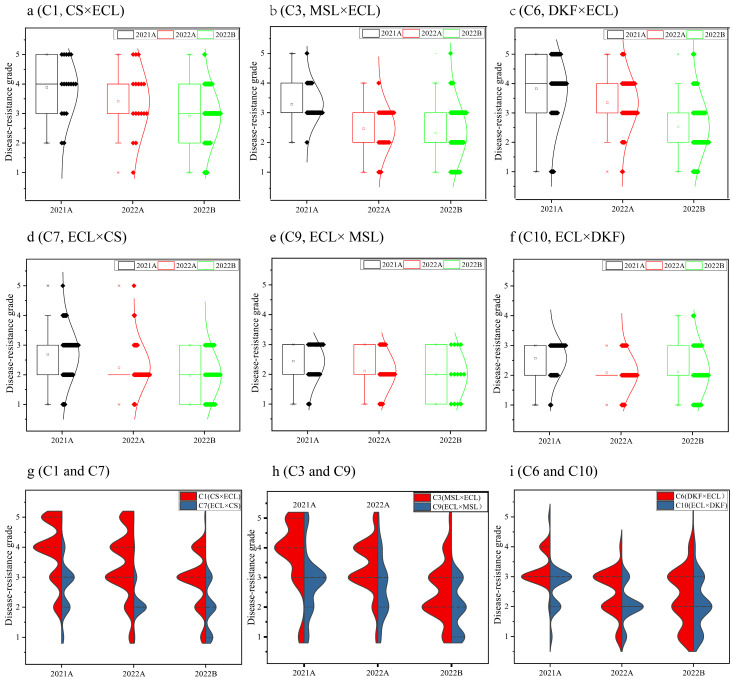
Genetic distribution of downy mildew resistance grades from field evaluations among progeny populations of different hybrid combinations. Note: (**a**–**f**): Distribution of downy mildew resistance grades among the progeny of six hybrid combinations; (**g**–**i**): Comparison of the distribution law of orthogonal and antigonal combinations. ECL, CS, MSL, and DKF represent varieties of Ecolly, Cabernet Sauvignon, Marselan, and Dunkelfelder, respecitively.

Genetic analysis of field downy mildew resistance grades was conducted on the progeny of six cross combinations. The results are presented in Figure 2. The progeny from different years and batches of hybrid combinations showed a typical normal distribution of downy mildew resistance grades. This finding demonstrates the quantitative genetic characteristics controlled by multiple genes, except for the 2021A C6 and C10 combinations. A significant difference was observed in the normal distribution of progenies between different years for the same hybrid combination; the downy mildew resistance grades in 2022A skewed more toward higher resistance than in 2021A. The genetic distribution trend of downy mildew resistance grades remained consistent for the same hybrid combination in the 2022A and 2022B batches, except for the C6 combination. Progeny derived from crosses where Ecolly was used as the maternal parent (e.g., C7 vs. C1, C9 vs. C3, and C10 vs. C6) consistently showed higher downy mildew resistance, suggesting a notable maternal inheritance effect. Progeny from the hybrid combinations of Ecolly and Dunkelfelder (C6 and C10) showed higher downy mildew resistance than those from Ecolly and Cabernet Sauvignon (C1 and C7) and Ecolly and Marselan (C3 and C9). These results suggest that C10, with Ecolly as maternal parent and Dunkelfelder as the paternal parent, may be the best choice among the six combinations.

In vitro downy mildew DI was identified for 4 parents and 1657 hybrid progenies over two consecutive years (Table 2). A significant difference was found in the downy mildew DI of the four parents. Their DIs were lower in 2022 than in 2021, and this ranking was consistent across different years: Ecolly < Dunkelfelder < Marselan < Cabernet Sauvignon. The range, average, and CV of the downy mildew DI varied considerably among the progeny of different hybrid combinations. Progeny from combinations with Ecolly as the maternal parent exhibited a lower downy mildew DI average than those with Ecolly as the paternal parent; moreover, the distribution ranges was smaller. For the same hybrid combination, the CV for the downy mildew DI in the 2021A progeny was lower than that of 2022A. Significant differences in H^2^ of the downy mildew DI were found among the progeny of different hybrid combinations.

### 2.3. Regression Analysis and Comprehensive Evaluation of Disease Resistance Traits

**Figure 3:**
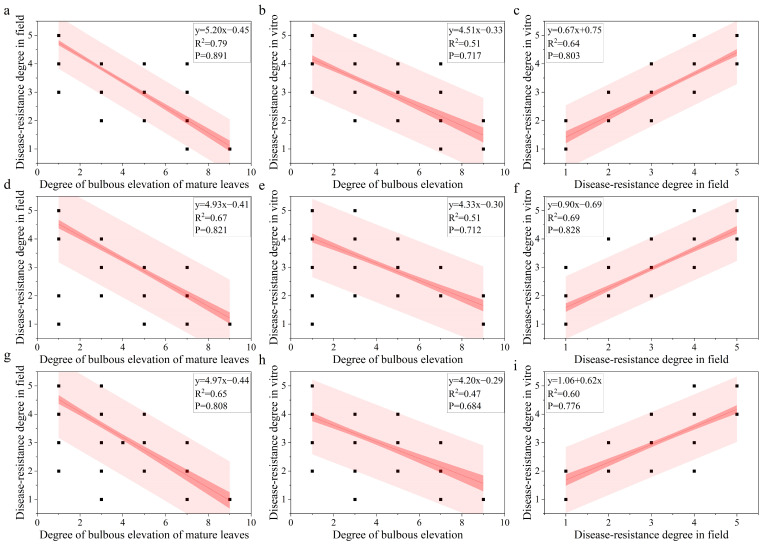
Regression analysis of leaf bulbous elevation, downy mildew resistance grade in the field, and downy mildew resistance grade in vitro. Note: (**a**–**c**): Population A in 2021; (**d**–**f**): Population A in 2022; (**g**–**i**): Population B in 2022.

A linear regression analysis was conducted to clarify the relationship among leaf bulbous elevation, field downy mildew resistance, and in vitro downy mildew resistance (Figure 3). The leaf bulbous elevation negatively correlated with both the field and in vitro downy mildew resistance grade. Its correlation coefficient with the field downy mildew resistance grade was higher than with in vitro for the same batch. The field downy mildew resistance grade positively correlated with the in vitro downy mildew resistance grade, with a correlation coefficient exceeding 0.6. A significant difference was found in the correlation between leaf bulbous elevation and the field downy mildew resistance grade within the same hybrid population across the two years. The correlation coefficient between the degree of leaf bulbous elevation and field downy mildew resistance grades was higher for the 2021A progeny than for the 2022A progeny. In summary, the magnitude of the correlation coefficients between different traits is affected by the year but remains stable within the same population.

**Figure 4:**
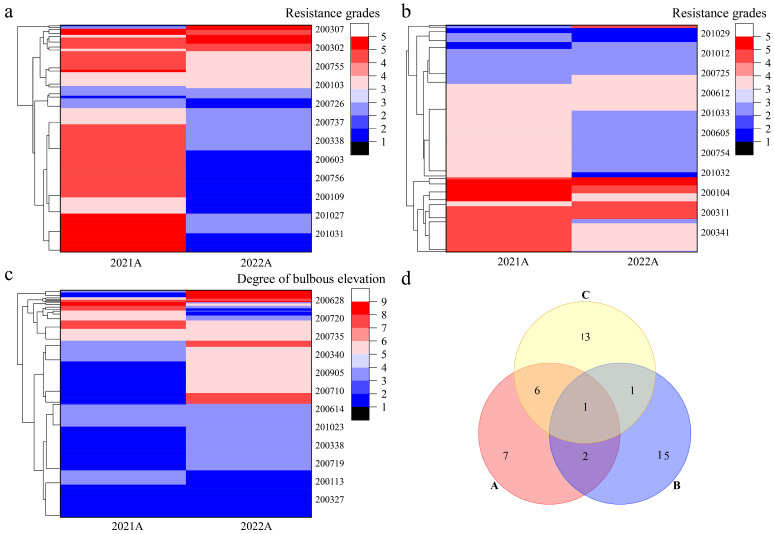
Clustering and Venn analysis of disease resistance traits in Population A. Note: (**a**): Cluster analysis of field disease resistance grades; (**b**): Cluster analysis of in virto disease resistance grades; (**c**): Cluster analysis of bulbous elevation degree; (**d**) Screening of highly resistant strains, A: field disease resistance; B: in vitro disease resistance; C: degree of leaf bulbous elevation.

**Figure 5:**
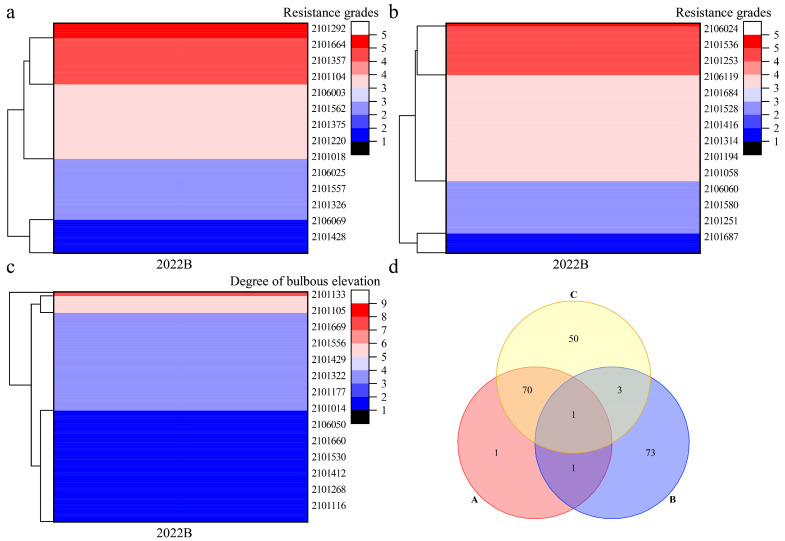
Clustering and Veen analysis of disease resistance traits in Population B. Note: (**a**): Cluster analysis of field disease resistance grades; (**b**): Cluster analysis of in virto disease resistance grades; (**c**): Cluster analysis of bulbous elevation degree; (**d**) Screening of highly resistant strains, A: field disease resistance; B: in vitro disease resistance; C: degree of leaf bulbous elevation.

Cluster analysis was performed on the disease resistance traits of Population A for two consecutive years (Figure 4) and Population B in 2022 (Figure 5). In Population A, field disease resistance clustered into five categories, with 16 strains exhibiting the highest resistance (Figure 4a); in vitro disease resistance was clustered into six categories, with 19 strains exhibiting the highest resistance (Figure 4b). The degree of leaf bulbous elevation clustered into four categories, with 21 strains exhibiting the highest resistance (Figure 4c). Venn analysis was performed on the strains with high levels of disease resistance across all three traits (Figure 4d). The following were identified: three strains with high levels of both field and in vitro disease resistance, suggesting consistency between disease resistance in the field and in vitro; seven strains with high levels of field disease resistance and a high degree of leaf bulbous elevation, suggesting that leaf bulbous elevation might be a self-reaction of the grapevines to downy mildew; two strains with high levels of in vitro disease resistance and a high degree of leaf bulbous elevation; and one strain with high levels of disease resistance for all three traits. In Population B, field disease resistance clustered into five categories, with 73 strains exhibiting the highest resistance (Figure 5a). In vitro disease resistance also clustered into five categories, with 78 strains showing the highest resistance (Figure 5b). The degree of leaf bulbous elevation was clustered into five categories, with 124 strains showing the highest resistance (Figure 5c). Venn analysis was performed on the strains with high disease resistance across three traits (Figure 5d). Among these, two strains showed high levels of both field and in vitro disease resistance. Moreover, 71 strains exhibited high levels of field disease resistance and a high degree of leaf bulbous elevation. Meanwhile, four strains were characterized by both high disease resistance in vitro and a high degree of leaf bulbous elevation. Notably, one strain exhibited high disease resistance across all three traits. These results indicate that the five strains with high disease resistance in both field and in vitro conditions can be used as parental materials for further recurrent hybridization, and that leaf bulbous elevation can be used as an auxiliary indicator for identifying disease resistance in the F2 progeny.

## 3. Discussion

### 3.1. Evaluation of Downy Mildew Resistance in V. vinifera

*V. vinifera* varieties are generally considered susceptible to disease, varying only in degree. Thus, fewer studies have been reported on disease resistance in *V. vinifera* varieties. However, Li et al. [25] identified downy mildew resistance in 140 *V. vinifera* varieties and powdery mildew resistance in 68 *V. vinifera* varieties. The results indicated significant differences in disease resistance among *V. vinifera* varieties. The resistance of *V. vinifera* varieties to downy mildew and powdery mildew exhibited a continuous distribution. The downy mildew resistance of four parents was identified by in vitro inoculation and two consecutive years of field investigation. Results showed that Ecolly had the lowest DI, indicating the highest resistance, followed by Dunkelfelder, Marselan, and Cabernet Sauvignon. This finding is consistent with previous reports on downy mildew resistance in *V. vinifera* varieties [26]. Temperature, humidity, and rainfall are key factors in downy mildew prevalence [27]. Downy mildew thrives under field conditions with relatively low temperatures (20 °C), continuous rainfall, and high air humidity (>85%) [28,29]. In the Yangling area, downy mildew generally first appears in mid-May. Its peak outbreak typically occurs in late July and continues until September, when it starts to decline [2]. Low rainfall during the peak disease period can delay the peak period of downy mildew, leading to its decline due to the maturity of grape leaves. This factor explains the lower overall downy mildew DI in 2022 than in 2021.

### 3.2. Genetic Characteristics of Disease Resistance in V. vinifera Hybrid Progeny

Grape resistance to pathogens may be genetically characterized across populations. For example, powdery mildew resistance inheritance varies. It has been described as a quantitative trait inheritance in *V. vinifera* and *Vitis davidii* [30,31]. In Chinese wild grapes [32], it is a dominant independent inheritance controlled by multiple genes. In *Vitis rotundifolia Michx.*, it is a trait dominantly inherited under single-gene control [33]. Other grape diseases, such as Downy mildew, grey mould, and spot anthracnose, are all quantitative traits controlled by multiple genes [34,35]. Downy mildew resistance in the F1–F4 progeny of the interspecific hybrid *Vitis amurensis Rupr.* exhibits a continuous distribution, favouring the resistant parent. Hybrid combinations with more resistant parents yielded a higher number of resistant strains. This finding suggests that resistance is a quantitative trait inheritance controlled by multiple genes; in addition, the disease resistance genes exert a cumulative, dominantly inherited effect on the progeny [36]. Downy mildew resistance in *V. vinifera* is also considered a quantitative trait. Its control by micro-efficient genes and their constant accumulation leads to superparental inheritance in *V. vinifera* intraspecific hybrid progeny [22,23]. This occurrence was also confirmed in the present study: downy mildew resistance in the *V. vinifera* intraspecific hybrid progeny was continuous, showing a skewed normal distribution and a degree of superparental inheritance, mostly expressed as convergent variation, consistent with the characteristics of quantitative inheritance. Bias statistics and genetic analysis indicated differences among hybrid progeny populations. Progeny populations from inverse combinations exhibited lower disease resistance grades than those from orthogonal combinations, suggesting maternal advantage. However, no completely biassed distribution was observed among orthogonal and backcross populations. The downy mildew resistance of the progeny was strongly influenced by genetics and demonstrated high stability across different years.

### 3.3. Relationship Between Disease Resistance Traits

Leaf morphology correlates with disease resistance. As previously reported, dorsal leaf villi can resist downy mildew infection on grape leaves to a certain extent. This finding suggests that the downy mildew resistance of a grape variety can be intuitively and easily determined from its leaf hair density index [37]. Moreover, the stomatal density of the leaf surface exhibits a significant positive correlation with the downy mildew susceptibility index. The regression coefficient is significant, implying that it can be directly used as an indicator of downy mildew resistance [38]. The bulbous elevation of leaves is considered a natural barrier to pathogen infection. Moreover, it may be a symptom of grapevine leafroll disease, which can weaken plant growth and reduce yield and quality [39,40]. The current study found a negative correlation between the degree of leaf bulbous elevation and the downy mildew resistance grade. This finding indicates that the higher the degree of leaf bulbous elevation, the higher the downy mildew resistance. The correlation coefficient between the degree of leaf bulbous elevation and the field resistance grade was higher than its correlation coefficient with the in vitro resistance grade. This observation suggests that it can potentially be used as a reference index for identifying field downy mildew resistance. It may relate to the process by which downy mildew infects leaves: leaves in the field develop a certain defence after pathogen infestation, thereby enhancing the degree of leaf bulbous elevation; meanwhile, the degree of leaf bulbous elevation remains unchanged in vitro [2]. More trait indicators are needed to improve the disease resistance evaluation system. Additionally, due to the influence of unpredictable climatic factors in the field, the degree of correlation may fluctuate, and the assessment of disease resistance in the same batch of offspring often requires data from multiple years of investigation. A significant positive correlation was determined between the field and in vitro resistance grades, consistent with previous research [41]. Therefore, field and in vitro identification results can be mutually complementary. For samples where external environmental conditions are not completely consistent, downy mildew resistance can be identified in vitro.

### 3.4. Screening of Hybrid Progeny with High Downy Mildew Resistance

The establishment of correlations between disease resistance traits may reduce the number of traits to be evaluated in future genotypes or progeny. In addition, understanding the correlation between these traits is crucial, given that improvement in one trait may adversely affect other traits [42]. In the present study, clustering and Venn analysis were conducted based on the field disease resistance grade, in vitro disease resistance grade, and degree of leaf bulbous elevation. The results showed 229 progeny groups identified in Population A, including 16 strains with high field resistance, 19 strains with high in vitro resistance, and 21 strains with a high degree of leaf bulbous elevation. In addition, 1428 progeny were identified in Population B, including 73 strains with high field disease resistance, 78 strains with in vitro disease resistance, and 124 strains with a high degree of leaf bulbous elevation. Among them, two strains showed the three disease resistance traits, but one exhibited weak growth. Venn analysis also screened five strains with high levels of both field and in vitro disease resistance. After one strain was removed because of weak growth, the remaining four strains were designated as key candidates for future research.

## 4. Materials and Methods

### 4.1. Materials

**Figure A1:**
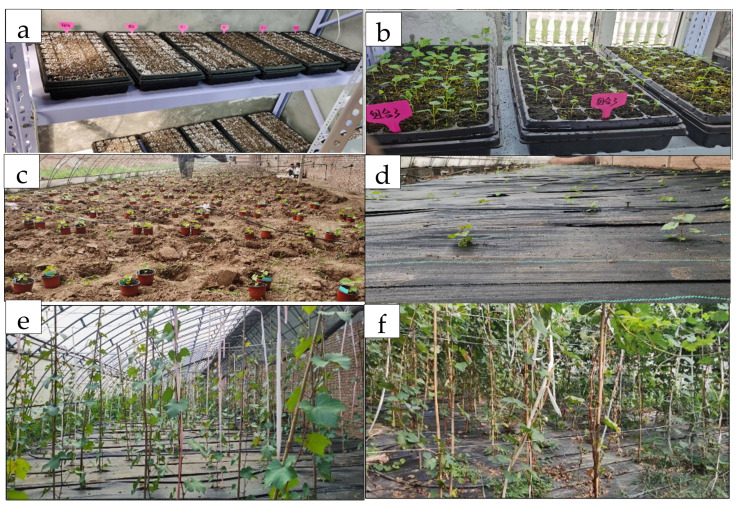
Hybrid progeny development: hole tray sowing (**a**), greenhouse seedling refining (**b**), field planting (**c**,**d**), and growth at the time of survey (**e**,**f**).

A total of four cultivars of *V. vinifera* were selected as parents, including Ecolly, a disease-resistant, high-quality variety independently bred by intraspecific recurrent selection in *V. vinifera* (IRSV), Cabernet Sauvignon, Marselan, and Dunkelfelder. The entire hybridization procedures and hybrid seed germination are described in previous research reports [43]. The three orthogonal combinations and their corresponding three inverse combinations are presented in Table 3. These combinations include 229 hybrids from 6 combinations in 2020, planted in April 2021 (Population A), and 1428 hybrids from 6 combinations in 2021, planted in April 2022 (Population B). All progenies were cultivated in April 2021 and April 2022 at the Yangling Shengtang Winery of Shaanxi Province (lat. 34° N, long. 108° E) (Figure A1). This area has a semiarid, continental monsoon climate, and the soil type is bauxite. The main disease threat to grape cultivation is downy mildew, caused by *Plasmopara viticola*. Rows were oriented west–east, with a vine spacing of 0.6 m between vines and 1.0 m between rows. All tested materials (parents and progeny) were maintained under the same conditions, including pruning, irrigation, soil management, and fertilization. Basic disease control included spraying a 6% kaolin particle film solution from June through August annually [3].

### 4.2. Investigation of the Degree of Leaf Bulbous Elevation

The botanical traits were investigated in cuttings of 4 parents and F1 progeny seedlings from 6 combinations, respectively, from 2021 to 2022. This method adhered to the specification for grape germplasm description [44]. The degree of leaf bulbous elevation on the upper surface of adult leaves was classified into 5 levels, no or very weak, weak, medium, strong, and extremely strong, which correspond to 1, 3, 5, 7, and 9. Three biological replicates were set up for the investigation of the parents.

### 4.3. Identification and Grading of Downy Mildew

During the peak incidence period of downy mildew (August 2021 and September 2022), field investigations and in vitro identification experiments were performed on the 4 parents and their respective F1 progenies. Young, healthy, pest- and disease-free leaves were initially collected for inoculation in vitro, generally from the first 10 leaves of new shoots. A field survey was then conducted, covering the middle part of new shoots and avoiding the top and bottom 3 leaves, and the affected area was recorded.

For in vitro characterization, 5 complete leaves were selected from each progeny and brought back to the laboratory. After the leaves were sterilized, 30 leaf discs with a 15 mm internal diameter hole punch were taken, avoiding the major veins, and randomly distributed among three replicates. The bottom of a 90 mm diameter disposable Petri dish was lined with 2 layers of sterile filter paper to which 4 mL of sterile distilled water was added. Leaf discs were placed into the Petri dish, the underside facing upward. Subsequently, 10 leaf discs were randomly arranged in each Petri dish, with 3 Petri dishes set up for each F1 progeny as technical replicates. Hybrid parents were set up with 3 biological replicates.

Preparation and inoculation of the spore suspension involved collecting diseased fresh leaves of vines at the peak of field downy mildew incidence. Spots on the leaf surface were washed off with sterile water, and leaves were incubated for 24 h in an incubator at 22 °C, with 90% humidity, under dark conditions. When new sporangia grew on the diseased leaves, they were gently brushed into sterile distilled water. A sterile soft-bristle brush was used. The solution was shaken well, and the spore concentration was adjusted with a hemocytometer to 1 × 10^5^/mL using the hemocytometer method. Subsequently, 30 μL of spore suspension was added to the centre of each leaf disc. After inoculation, the Petri dishes were sealed with a parafilm and then placed in an artificial climate incubator at 20 ± 2 °C, with 90% relative humidity, under 12 h light/darkness conditions. After inoculation with downy mildew for 24 h, the spore suspension on the leaf discs was blotted dry with sterile filter paper, and incubation continued under the same conditions. The leaf discs inoculated with downy mildew for 7 d (168 h) were photographed with a digital camera. The percentage of the affected area was calculated using Photoshop CS5.

The percentages of the affected area on both leaves and leaf discs were classified using the Desaymard “0–10” classification method [45]. The grading system is as follows: Grade 0, no diseased spots; Grade 1, the infected area accounts for 0.1–5% of the whole leaf/disc; Grade 3, the infected area accounts for 6–25% of the whole leaf/disc; Grade 5, the infected area accounts for 26–50% of the whole leaf/disc; Grade 7, the infected area accounts for 51–75% of the whole leaf/disc; and Grade 9, the infected area accounts for 76–100% of the whole leaf/disc. The DIs for both field and in vitro leaves were calculated using Equation (1). The DI for each strain was then averaged across leaves or leaf discs.

(1) $$\mathrm{DI}=\frac{\sum \left(\mathrm{grade}\;\times \;\mathrm{numbers}\;\mathrm{of}\;\mathrm{infected}\;\mathrm{leaves}\;\mathrm{in}\;\mathrm{that}\;\mathrm{grade}\right)}{\mathrm{Total}\;\mathrm{leaf}\;\mathrm{numbers}\;\times \;\mathrm{highest}\;\mathrm{grade}}\;\times \;100$$

The degrees of downy mildew infection on leaves, both in the field and in vitro, were graded into five classes according to the standards of the International Board of Plant Genetic Resources (IBPGR) [46]. The system is as follows: Grade 1, with a DI of 0, indicating immunity; Grade 2, with a DI of 0.1–5.0, indicating high resistance; Grade 3, with a DI of 5.1 to 25.0, indicating resistance; Grade 4, with a DI of 25.1–50.0, indicating susceptibility; and Grade 5, with a DI of 50.1% to 100%, indicating high susceptibility.

### 4.4. Data Analysis

The CV, ultra-high affinity ratio (HH), super-relative median ratio (HM), low–low affinity ratio (LL), and generalized heritability (H^2^) were calculated based on Equations (2)–(6):

(2) $$\mathrm{CV}(\%)\;=\;\frac{Standard\;deviation\;of\;hybrid\;offspring}{F}\;\times \;100$$

(3) $$\mathrm{HH}(\%)=\frac{\mathrm{Number}\;\mathrm{of}\;\mathrm{strains}\;\mathrm{higher}\;\mathrm{than}\;\mathrm{the}\;\mathrm{higher}\;\mathrm{parent}}{\mathrm{Number}\;\mathrm{of}\;\mathrm{the}\;\mathrm{test}\;\mathrm{strains}}\;\times \;100$$

(4) $$\mathrm{HM}(\%)=\frac{\mathrm{Number}\;\mathrm{of}\;\mathrm{strains}\;\mathrm{higher}\;\mathrm{than}\;\mathrm{the}\;\mathrm{median}\;\mathrm{of}\;\mathrm{parents}}{\mathrm{Number}\;\mathrm{of}\;\mathrm{the}\;\mathrm{test}\;\mathrm{strains}}\;\times \;100$$

(5) $$\mathrm{LL}(\%)=\frac{\mathrm{Number}\;\mathrm{of}\;\mathrm{strains}\;\mathrm{lower}\;\mathrm{than}\;\mathrm{the}\;\mathrm{low}\;\mathrm{parent}}{\mathrm{Number}\;\mathrm{of}\;\mathrm{the}\;\mathrm{test}\;\mathrm{strains}}\;\times \;100$$

(6) $$H^{2}=\frac{\left[(V_{H}-1/2(V_{P1}+V_{P2})\right]}{V_{H}}\;\times \;100$$

For phenotype variance calculation, V_H_ represents the phenotype variance of the hybrid progeny population. V_P1_ corresponds to the phenotype variance of the female parent. V_P2_ denotes the phenotype variance of the male parent.

Microsoft Excel was used for data collection. SPSS 22.0 was employed for statistical and significance analysis. Data were analyzed for significance via one-way ANOVA with Duncan’s multiple comparisons at a significance level of 0.05. Genetic curve fitting, regression and correlation analysis, and illustration were performed using Origin Pro 2021.

## 5. Conclusions

In conclusion, significant differences in downy mildew resistance were observed among grape varieties. This resistance is inherited as a quantitative trait, controlled by multiple genes, and conforms to a skewed normal distribution with maternal dominance to a certain extent. The degree of leaf bulbous elevation negatively correlated with the downy mildew resistance level of leaves. Combined with vegetative growth, leaf bulbous elevation can serve as an early indicator for screening downy mildew resistance. Intraspecific hybridization of *V. vinifera* varieties yields resistant progeny. This study successfully identified five such strains with high disease resistance under both field and in vitro conditions. These strains can potentially represent new germplasm for disease resistance breeding.

## Figures and Tables

**Table 1:** Field investigation results and bias statistics for downy mildew disease index in leaves of various hybrid combinations.

Year	Hybrid Combinations (♀ × ♂)	Disease Index of Parents			Bias in Progeny			CV (%)	H^2^
		P1 (♀)	P2 (♂)	$\bar{P}$	HH (%)	HM (%)	LL (%)		
2021A	C1 (CS × ECL)	51.85 ± 8.42	7.39 ± 2.75	29.62	70.59	88.24	0	49.60	67.23
	C7 (ECL × CS)	7.39 ± 2.75	51.85 ± 8.42		13.85	70.77	7.81	62.92	63.60
	C3 (MSL × ECL)	48.14 ± 5.62	7.39 ± 2.75	27.77	71.93	91.23	5.26	58.06	76.65
	C9 (ECL × MSL)	7.39 ± 2.75	48.14 ± 5.62		25.93	59.26	7.41	51.42	65.21
	C6 (DKF × ECL)	21.40 ± 4.89	7.39 ± 2.75	14.40	28.57	96.43	0	35.86	62.14
	C10 (ECL × DKF)	7.39 ± 2.75	21.40 ± 4.89		33.33	76.19	7.14	46.62	59.52
2022A	C1 (CS × ECL)	47.22 ± 9.22	4.26 ± 1.45	25.74	47.06	88.24	5.88	63.76	76.73
	C7 (ECL × CS)	4.26 ± 1.45	47.22 ± 9.22		12.5	29.69	6.25	62.86	62.09
	C3 (MSL × ECL)	33.33 ± 5.62	4.26 ± 1.45	18.80	43.86	91.23	3.51	51.92	65.88
	C9 (ECL × MSL)	4.26 ± 1.45	33.33 ± 5.62		14.81	40.74	14.81	77.45	78.92
	C6 (DKF × ECL)	9.09 ± 3.28	4.26 ± 1.45	6.68	28.57	64.29	10.71	59.34	59.47
	C10 (ECL × DKF)	4.26 ± 1.45	9.09 ± 3.28		11.43	37.14	14.29	57.86	57.39
2022B	C1 (CS × ECL)	47.22 ± 9.22	4.26 ± 1.45	25.74	25.01	81.68	8.4	61.90	58.89
	C7 (ECL × CS)	4.26 ± 1.45	47.22 ± 9.22		11.76	62.75	39.22	84.26	55.23
	C3 (MSL × ECL)	33.33 ± 5.62	4.26 ± 1.45	18.80	12.74	66.41	27.41	88.34	68.82
	C9 (ECL × MSL)	4.26 ± 1.45	33.33 ± 5.62		15.38	61.54	30.76	78.44	79.99
	C6 (DKF × ECL)	9.09 ± 3.28	4.26 ± 1.45	6.68	22.39	52.99	8.21	64.62	57.46
	C10 (ECL × DKF)	4.26 ± 1.45	9.09 ± 3.28		15.56	55.56	24.44	77.92	61.23

Note: $\bar{P}$: parental mean; HH: ultra-high affinity ratio; HM super-relative median ratio; LL low–low affinity ratio; CV: coefficient of variation; H^2^: generalized heritability.

**Table 2:** In vitro identification and genetic variation in downy mildew disease index in vitro in different hybridization combinations.

Year	Hybrid Combinations (♀ × ♂)	Disease Index of Parents			Disease Index of Progeny		CV (%)	H^2^
		P1 (♀)	P2 (♂)	$\bar{P}$	Range	Average		
2021A	C1 (CS × ECL)	49.29 ± 6.87	9.40 ± 4.85	29.35	2.22–70.37	34.32 ± 14.32	57.07	66.83
	C7 (ECL × CS)	9.40 ± 4.85	49.29 ± 6.87		0–55.55	11.08 ± 11.05	99.70	60.90
	C3 (MSL × ECL)	48.21 ± 9.56	9.40 ± 4.85	28.81	0–88.89	42.89 ± 25.10	58.52	70.34
	C9 (ECL × MSL)	9.40 ± 4.85	48.21 ± 9.56		0–15.56	6.77 ± 5.10	75.30	56.31
	C6 (DKF × ECL)	29.40 ± 4.36	9.40 ± 5.36	19.40	0–55.56	21.92 ± 9.95	45.39	62.60
	C10 (ECL × DKF)	9.40 ± 5.36	29.40 ± 4.36		0–22.16	7.89 ± 5.81	73.64	61.91
2022A	C1 (CS × ECL)	29.12 ± 7.26	4.12 ± 1.36	16.62	0–70.37	35.31 ± 20.97	59.39	77.39
	C7 (ECL × CS)	4.12 ± 1.36	29.12 ± 7.26		0–55.55	6.14 ± 9.66	157.45	66.25
	C3 (MSL × ECL)	26.54 ± 4.13	4.12 ± 1.36	15.33	0–55.55	22.71 ± 15.48	68.18	79.72
	C9 (ECL × MSL)	4.12 ± 1.36	26.54 ± 4.13		0–22.22	4.52 ± 4.85	107.45	64.57
	C6 (DKF × ECL)	7.22 ± 1.78	4.12 ± 1.36	5.67	0–34.44	6.70 ± 7.50	107.31	76.26
	C10 (ECL × DKF)	4.12 ± 1.36	7.22 ± 1.78		0–11.08	3.22 ± 2.68	83.06	76.50
2022B	C1 (CS × ECL)	29.12 ± 7.26	4.12 ± 1.36	16.62	0–86.67	15.28 ± 12.98	85.00	61.71
	C7 (ECL × CS)	4.12 ± 1.36	29.12 ± 7.26		0–13.89	3.24 ± 3.54	109.17	53.00
	C3 (MSL × ECL)	26.54 ± 4.13	4.12 ± 1.36	15.33	0–63.89	9.02 ± 12.97	143.91	75.63
	C9 (ECL × MSL)	4.12 ± 1.36	26.54 ± 4.13		0–19.44	4.76 ± 5.95	124.87	62.60
	C6 (DKF × ECL)	7.22 ± 1.78	4.12 ± 1.36	5.67	0–53.09	8.55 ± 10.19	119.13	82.27
	C10 (ECL × DKF)	4.12 ± 1.36	7.22 ± 1.78		0–30.56	5.77 ± 7.55	130.89	76.24

**Table 3:** Hybrid combinations and progeny.

Population	Orthogonal Combinations	Correspondence Number	Inverse Combinations	Correspondence Number
A	C1 (CS × ECL)	2001001–2001017	C7 (ECL × CS)	2007001–2007064
	C3 (MSL × ECL)	2003001–2003057	C9 (ECL × MSL)	2009001–2009027
	C6 (DKF × ECL)	2006001–2006029	C10 (ECL × DKF)	2010001–2010035
B	C1 (CS × ECL)	2101001–2101726	C7 (ECL × CS)	2107001–2107051
	C3 (MSL × ECL)	2103001–2103459	C9 (ECL × MSL)	2109001–2109013
	C6 (DKF × ECL)	2106001–2106134	C10 (ECL × DKF)	2110001–2101045

Note: Population A is a hybrid population generated in 2020 and cultivated in April 2021; Population B is a hybrid population generated in 2021 and cultivated in April 2022. CS, Cabernet Sauvignon; ECL, Ecolly; MSL, Marselan; DKF, Dunkelfelder.

## Data Availability

The raw data supporting the conclusions of this article will be made available by the authors on request.
